# Current and Future Advances in the Detection and Surveillance of Biosecurity-Relevant Equine Bacterial Diseases Using Loop-Mediated Isothermal Amplification (LAMP)

**DOI:** 10.3390/ani13162663

**Published:** 2023-08-18

**Authors:** Alexandra Knox, Gemma Zerna, Travis Beddoe

**Affiliations:** Department of Animal, Plant and Soil Sciences, Centre for AgriBioscience, La Trobe University, Bundoora, VIC 3082, Australia; a.knox@latrobe.edu.au (A.K.); g.zerna@latrobe.edu.au (G.Z.)

**Keywords:** equine, bacteria, loop-mediated isothermal amplification, monitoring, chemical additives

## Abstract

**Simple Summary:**

Bacterial infections continue to cause ongoing health problems in the global equine industry, resulting in substantial loss of valuable economic contributions worldwide. Biosecurity-relevant equine bacterial diseases have resulted in restricted importation and exportation of horses, impediment of breeding, and cancellation of culturally important events. Vigilant, rapid diagnostics and surveillance of such diseases are vital to prevent outbreaks and to provide timely management and treatment strategies. However, current methodologies are outdated and, at times, unreliable. This review outlines how the modern technique of loop-mediated isothermal amplification can aid in the protection of the equine industry, and the current advancements further its ability for in-field deployment.

**Abstract:**

Horses play an important role throughout the world, whether for work, culture, or leisure, providing an ever-growing significant contribution to the economy. The increase in importation and movement of horses, both nationally and internationally, has inevitably allowed for the global equine industry to grow. Subsequently, however, the potential for transmission of fatal equine bacterial diseases has also escalated, and devasting outbreaks continue to occur. To prevent such events, disease surveillance and diagnosis must be heightened throughout the industry. Current common, or “gold-standard” techniques, have shown to be inadequate at times, thus requiring newer technology to impede outbreaks. Loop-mediated isothermal amplification (LAMP) has proven to be a reliable, rapid, and accessible tool in both diagnostics and surveillance. This review will discuss equine bacterial diseases of biosecurity relevance and their current diagnostic approaches, as well as their respective LAMP assay developments. Additionally, we will provide insight regarding newer technology and advancements associated with this technique and their potential use for the outlined diseases.

## 1. Introduction

The equine industry, an enduring and essential entity worldwide, plays a crucial role in diverse cultures and communities by serving as a source of employment, entertainment, and companionship and providing substantial economic value of an estimated USD $300 billion annually [1,2,3]. The use of horses in various sectors, such as farming and entertainment, has been established for some time and is continuously expanding, for example, involvement in human psychotherapy [4,5,6,7]. With such an expansive range of contributions, it is essential to keep this industry stable and growing. The movement and trade of horses have rapidly increased worldwide over the past several decades, which has created more opportunities for importation and exposure of diseases, subsequently applying pressure on current biosecurity management practices [1,8]. Whilst these measures throughout countries are diligent and ever-adapting to new situations, there remains an apparent urgency for improved surveillance techniques to prevent detrimental disease outbreaks [3,9,10]. For a vast number of bacterial diseases of concern, current diagnostics and surveillance methodology rely on out-of-date technology or are time-consuming with a lengthy turnaround of results [11,12,13]. In addition to international movement and trade, surveillance at equine events and on farms should remain vigilant; this requires accessible technology that can be utilized in a range of environments, including resource-poor communities. Therefore, continuous and rigorous monitoring and detection methods should be of the upmost importance for equine research [3,14].

Whilst attention is largely focused on controlling viral pathogens [15,16], bacterial disease poses similar damaging outcomes [17,18]. Many highly contagious bacterial diseases can be transmitted easily from direct contact between horses or indirect contact with fomites. Despite extensive biosecurity laws for the importation and exportation of horses worldwide, bacterial outbreaks continue to frequently occur [8,19]. In turn, countries considered ‘disease-free’ of specific pathogens can quickly become endemic, even starting from a single importation of an infectious horse [20,21,22]. This has been the case in the United States with contagious equine metritis (CEM), a virulent venereal disease in horses. An initial outbreak in Kentucky in 1978 saw a significant economic impact to their equine industry, particularly for Thoroughbreds [23,24,25]. Following this, the US experienced further smaller outbreaks in 1979 [26], 1982 [23], and 2006 [27] in Missouri, Kentucky, and Wisconsin, respectively. These outbreaks were all related to the importation of a single infected horse [23,25,26,27]. Fortunately, these outbreaks were small-scale, and the disease was eradicated with proper measures, enabling the US to regain its CEM-free status [23]. However, in 2008, Kentucky had a substantial outbreak occur involving the shipment of infected semen. Contaminated equipment used for artificial insemination caused a large uncontrollable outbreak [28,29,30]; consequently, the US has not been able to reclaim a CEM-free status [22,23]. This is just one of many similar devastating bacterial outbreaks that have been seen around the world.

Reliable and rapid diagnostic and surveillance procedures are a key factor for aiding in the prevention of outbreaks [31,32]. However, current diagnostic techniques for numerous equine bacterial diseases of concern rely on traditional methods, such as bacterial cell culture. These techniques are time-consuming and laborious, often requiring days to weeks to obtain results [10,11,32]. Additionally, many studies have noted a low level of sensitivity and frequent reporting of false-positive or false-negative results [32,33]. Routine methods such as serological assays may provide more reliable results; however, as these methods rely on the detection of antibodies, a secondary test is recommended to confirm an active infection [32,34]. To overcome sensitivity issues, polymerase chain reaction (PCR) tests have been implemented for many equine bacterial diseases [35,36,37]. However, the requirement of expensive and large machinery prevents widespread use in diverse and unequipped environments [3,16,35]. In contrast, loop-mediated isothermal amplification (LAMP) has shown to be a rapid nucleic acid amplification technique that is able to provide results in under 20 min whilst maintaining reliability, sensitivity, and specificity comparable to PCR-based methods. Additionally, this technology is designed to be field-deployable, eliminating the need to transport either the animal or collected samples, thus reducing the delay between sample collection and result confirmation. This, in turn, can provide the horses with appropriate treatment in a timely matter to mitigate infections early [38,39]. This assay has already been implemented for many viral [40,41,42,43], bacterial [44,45,46], and parasitic [47,48] equine diseases. Owing to the advantages of this method, continuous research and development has been made to further enhance both the technology itself and for accessible and accurate monitoring methods [39,49].

This review focuses on equine bacterial pathogens of biosecurity importance, as well as traditional and gold-standard techniques utilized for disease detection and surveillance. Additionally, we discuss the current use of LAMP in equine bacterial disease monitoring and how continuous advances in incorporating modern technology can aid in outbreaks, emphasizing the implementation of routine surveillance across an assortment of environments and settings.

## 2. Equine Bacterial Diseases of Biosecurity Relevance

Equine bacterial disease outbreaks are prominent worldwide and continue to have damaging impacts on the industry. In addition to the effect on the health and well-being of animals, outbreaks can cause restrictions on horse importation and exportation and cancelation of socially and economically important events [20,50,51,52]. Several bacterial diseases pose an imminent risk to biosecurity due to ease of transmission, particularly in areas with a high rate of movement [1,10]. This section will outline four examples of bacterial diseases of biosecurity importance and detail their clinical signs, transmission, diagnostics, control, and prevention. Additionally, we will highlight biosecurity risks for each pathogen and where improvements to routine diagnostics should be made.

### 2.1. Rhodococcus Equi (Pneumonia)

*Rhodococcus equi* (*R. equi*), a soil actinomycete, is a pulmonary pathogen that induces pneumonia in horses, particularly foals. *R. equi* has been documented since 1923 as an animal pathogen [53], where it was originally categorized as a *Corynebacterium* before being identified as its own genus in 1977 [54,55,56]. Despite a substantial variation in the occurrence of cases reported in the literature, there is a consensus that avirulent *R. equi* can be frequently found at every equine farm, in both the environment and feces. Moreover, as current circulating strains commonly cause subclinical infections, many cases often go undiagnosed; thus, the actual prevalence of *R. equi* cannot be definitively determined [55,57]. Horses with clinical manifestation will typically present with chronic suppurative bronchopneumonia associated with suppurative lymphadenitis, and severe cases may become fatal within several hours or days after initial respiratory signs. Cases may occur with concurrent enteric disease, including septic or non-septic arthritis, hepatic and renal abscessation, or osteomyelitis; however, this is less common [57,58]. Foals are not the only equine population susceptible to *R. equi* and the associated complications; both mature and immunocompromised horses can experience pneumonia and enteritis [55,57]. Due to the environmental robustness of this bacterium, transmission can readily occur through aerosols on farms with low soil moisture, high temperatures, and scarce pasture growth [59,60,61]. Despite this, routine surveillance options are limited to blood testing on endemic farms [55,62]. The American Association of Equine Practitioners (AAEP) recommends either bacterial culture or PCR testing for a definitive diagnosis [63]]. Serological detection can be ineffective due to equine populations harboring residual antibodies against this pathogen as a result of continuous seroprevalence [55,63]. There is debate amongst literature regarding the current fatality rate of *R. equi*; some authors state it has substantially lowered since the 1987 introduction of a specific antibiotic combination to treat infections [58,64,65]. However, others claim incidences of death remain high due to the emergence of antibiotic resistance strains in 1992 [66,67,68]. It is evident alternative treatment protocols are required. Despite multiple attempts, vaccine development has thus far been ineffective [55,69,70,71]. However, protection against *R. equi* infection through transfusion of hyperimmune plasma has shown promising results, although reports have varied [72,73,74,75]. This commercially available product has been shown to reduce the severity of disease but not mortality [74], yet farms rely on this method as a preventative measure [76]. According to Bordin et al. [77], utilizing this prophylactic management is the current most efficacious option for prevention. However, Bordin et al. [77] reiterate that screening equine disease status is one of the most important and effective tools for prevention.

### 2.2. Streptococcus Equi Subspecies Equi (Strangles)

One of the most contagious bacterial diseases of horses is the acute upper respiratory disease caused by *Streptococcus equi* subspecies *equi*, commonly known as Strangles [78,79,80]. Predominantly affecting young foals and older horses, an infected individual can quickly become a chronic asymptomatic carrier that periodically sheds the bacterium and, thus, a potential source for an outbreak. Interestingly, Strangles has been referenced in literature dating back to the 13th century, being first formally described in 1888 [81,82]. This disease is prominent worldwide, causing outbreaks that can last up to 4 to 6 months and can become enzootic on farms [79,83]. There are multiple predisposing factors that can influence these outbreaks, such as overcrowding, a mixture of imported horses without adequate quarantine compliance, and various stressors, including travel, improper nutrition, weaning, and severe weather [83,84]. Unlike the closely related bacterium, *S. equi* subspecies *zooepidemicus*, Strangles are not present in the natural flora of the nasopharyngeal of equine; however, both can cause significant diseases [79,84]. Typically, a horse infected with Strangles will present with an acute fever, which may not subside until the horse is fully recovered, followed by the development of nasal discharge [78]. As the disease progresses, this nasal discharge will become mucopurulent, facilitating spread throughout the upper respiratory tract [78,79]. Subsequently, abscesses are formed on the submandibular lymph nodes and will develop to become firm and painful, which can cause tightening in the equine’s esophagus; hence, the term Strangles. These abscesses rupture a week to 10 days post onset of clinical signs but can take up to 4 weeks in some cases [85,86,87], with uncomplicated cases recovering at about 1–2 weeks post rupture [78]. Severe complications can occur, including guttural pouch empyema, purpura haemorrhagica, myositis, internal abscessation and further spread of the bacteria internally [79,85,88]. Nasal discharge and rupturing of abscesses contribute to transmission for both direct horse to horse contact, and indirect contact, such as contaminated equipment including housing, water and feed sources and gear [89,90,91]. Shedding of the bacteria can be persistent for a substantial period, most commonly horses remain infections for at least 6 weeks post discharge cessation [88,92]. However, it has been previously documented an infected horse continued to shed for 39 months post recovery, while this is a single case it is important to note the variability of shedding periods [79,93,94]. As previously stated, some horses may also become chronic asymptomatic carriers, resulting in a continual origin for outbreaks [11,95].

Definitive diagnosis currently requires a qPCR test [78,96]; however, sensitivity is dependent on the anatomical location of the sample and collection technique and the stage of infection [34,97]. Culturing was originally used for detection, but multiple studies reported the inadequacy of this method due to the variability of clinical sensitivity [45,96], which will be discussed further below. Treatment and prognosis are conditional to the stage of the disease; however, most horses will fully recovery with minimal supportive care [79,86]. As with *R. equi* infections, antibiotic use is debated amongst scientists [86,98]. Whilst studies state antibiotics reduce the duration of the disease and degree of discharge and secretions [98,99,100], others state incorrect use, such as incorrect therapeutic regimen, results in failed treatment and subsequent reinfection [21,83,101]. Yet, many countries have access to a vaccine, and despite low efficacy, it can reduce the severity of the disease [85,86,102]. Due to limited prevention and treatment options, as well as the high transmissibility of Strangles, control of an outbreak is strenuous. Current recommendations include quarantining the affected horse for the entire duration of the disease, followed by a further minimum of 4 weeks after clinical signs subside [80,91]. During this, isolated horses must have separate equipment, and it is highly recommended that farmers eliminate any cross-contamination by having separate clothing or prevent personnel from visiting healthy horses if they have been in contact with infected horses. Additionally, extensive cleaning is a priority during outbreaks, ensuring all potentially contaminated areas are disinfected and organic material, including feed and manure, is disposed of away from any present horses. Additionally, stalls and paddocks should be left empty for 2–3 weeks post quarantine [80,85,95]. Controlling an outbreak on a farm may not be successful for some time, particularly if new cases arise and consequently reset the quarantine period, resulting in substantial loss of production or work, and thus income, on infected farms for an extensive period [78,85].

### 2.3. Taylorella Equigenitalis (Contagious Equine Metritis)

Contagious equine metritis (CEM) is a highly infectious venereal disease in horses caused by *Taylorella equingenitalis* (*T. equigenitalis*). Since its original discovery in 1977 in the United Kingdom [103], this equine-specific pathogen has spread throughout many countries, such as North and South America, Australia, and Japan [18], and is endemic throughout parts of Europe [104]. Typically, stallions are asymptomatic and thus remain as unidentified carriers that consequently transmit the disease to mares during mating [23,25,103]. For infected mares, symptoms can be severe and are generally localized throughout their reproductive tract, either impacting or entirely inhibiting breeding [20,104]. Symptoms commonly include uterine and vaginal inflammation, accompanied by thick, odorless discharge, which may be secreted within 1–3 days after mating. This discharge can accumulate in large amounts, which results in further discomfort and potential spread of infection in mares. Lesions may also form throughout the uterus and vagina during the course of the disease, and the animal may return to heat within a few days post-infection [20,105]. Most mares overcome the disease within 3–4 weeks post-infection; however, some may become chronic carriers and must be excluded from a breeding program entirely [22,106]. Fortunately, once identified, carriers can be treated by washing the external genitalia with disinfectants combined with antimicrobials [20,29,107]. Antibiotics may be used in some acute cases, but this is upon the recommendation of a veterinarian, as the optimal length of treatment is undefined [24,108]. Whilst the disease is not typically fatal, the period to naturally overcome the disease is unpredictable and may take several months or more. As a result, the infected mare will be excluded from breeding for an uncertain amount of time, incurring large economic losses for the farm [23,109]. After infection, full immunity is not acquired, and consequently, a mare can be infected multiple times in a short period of time, although symptoms are often more severe during the first infection [22,109].

In accordance with the World Organization for Animal Health (WOAH), formerly OIE, diagnosis relies on either isolation and identification of the bacteria or immunofluorescence antibody test (IFAT) and real-time PCR. Complement fixation testing (CFT) can be performed to detect an immune response; however, this is not considered a definitive result and must be supported using another means of diagnostic testing [110]. Control and prevention rely on screening of animals prior to any national or international movement, with positive cases requiring treatment until the animal tests negative and isolation can cease [22,109]. Identification of carriers is difficult despite being one of the most important aspects of control [111,112,113]. CEM outbreaks are periodic in the United States; however, in 2008, when a stallion tested positive during routine surveillance, an epidemiological investigation was initiated by the United States Department of Agriculture (USDA) [22]. This extensive investigation required testing of over 1,000 horses, returning 28 positive results. Each of these positive results could be traced to a single origin: a shared breeding facility in Kentucky, America. Where 23 stallions were infected through fomites and subsequently transmitted the disease through infected semen via artificial insemination or live breeding to 11 mares. This was the largest investigation in the US, affecting more than 48 states [13,20,23]. This outbreak highlights the cruciality of routine surveillance, as without these strict protocols this case may not have been identified, which potentially could have led to a much larger-scaled epidemic. Japan has previously demonstrated how consistent monitoring aids in successful eradication. CEM was first introduced to Japan in 1980, where it rapidly spread through the Thoroughbred population in the major breeding district of Hidaka-Iburi. By the end of the year, three hundred and twenty-one horses were diagnosed with the disease [109,114,115]. In 1999 Anzai et al. [116] developed a PCR test that was implemented in a surveillance program for Japan, which began in 2001. This involved testing all Thoroughbred horses involved in breeding programs before the season began. Each horse breeding pair had to undergo three separate PCR tests and were consequently excluded from breeding programs for three years if they returned a positive result [109,117]. Five years later, in 2006, Japan was no longer detecting any CEM cases, officially declaring their successful eradication in 2010 [109,118]. This highlights the important role diagnostics and surveillance play in mitigating bacterial disease outbreaks, as well as aiding in potential elimination.

### 2.4. Burkholderia Mallei (Glanders)

Glanders, caused by the zoonotic pathogen *Burkholderia mallei* (*B. mallei*), is rapidly becoming an increasing concern for the equine industry. Despite eradication from much of the Western world in the early 20th century, there has been a re-emergence of cases over the past ten to twenty years [119,120]. Additionally, Glanders is now causing incidences in regions that previously did not harbor the disease. This could be owed to several factors, such as the high contagious nature of the bacteria, the potential for humans to be an incidental host, and resistance to current treatment options [17,119,120]. The severity of this disease has resulted in the United States classifying *B. mallei* as a tier 1 biological agent [119,121]. Glanders typically present as either acute or chronic infections in equids, with the latter being more common in horses [122]. Despite being classified as chronic, infections in horses are frequently described as “acute episodes” where an infected horse appears to be recovering, only to succumb to the disease quickly thereafter [12,120]. There are three sub-forms of chronic infection in horses: nasal, pulmonary, and cutaneous (often referred to as farcy). Horses infected with the nasal form can present with nasal discharge, either uni- or bilateral, with thick yellow mucous, which causes crusting around the nostrils. Large ulcers can form on the nasal septum that eventually heal but leave scarring. Lymph nodes may swell; however, they are generally nonpainful and resolve without treatment. The pulmonary form is often considered an extension to the nasal form, where infection migrates to the lower respiratory tract, causing abscessation within the lung parenchyma and infrequently in the liver and spleen. Additionally, an infected horse might develop painful ulcers on the trachea [120,123]. Cutaneous infections cause ulceration around the muzzle and limbs and are often accompanied by thick discharge. The majority of cases will also involve lymph node vessels becoming enlarged with a corded-like appearance, hence the term “farcy”. Progression of all forms can take months to even years before death, in which a horse remains infectious and can be a potential source of an outbreak throughout [120,124]. Humans, on the other hand, generally present with upper or lower respiratory symptoms, where localized infection can occur and potentially develop into septicemia or involve an acute pulmonary infection, frequently resulting in death [125,126]. Despite extensive studies into this disease, the possibility of horse-to-horse transmission is still debated. According to the WOAH, the most common source is through ingestion of contaminated feed and water or via direct contact with an open wound, lesion, or through the mucosa [127]. Whilst vertical transmission from mare to foal is noted, transmission from stallions to mares is less common [17]. Unlike horses, human-to-human transmission is considered rare and is mainly limited to direct contact with lacerated skin or the mucosal membrane or from contact with an infected animal. Despite the debate on aerosol transmission for equine infections, it is agreed that this mode of transmission in humans can occur [125,128].

While the exact mortality rate in horses is undetermined, in humans, there is a consensus it is as high as 95% without proper treatment [129,130]. Yet, for both species, there is no available vaccine or approved treatment regimen, as antibiotics are inefficacious due to resistance [124,125]. Owing to the severity of the disease and zoonotic potential, governments have implemented a test and slaughter policy for infected horses, including the U.S., where treatment is prohibited [17,131]. Control of outbreaks within equine facilities is difficult and often unsuccessful, relying on basic measures of surveillance, quarantine, and extensive cleaning following euthanasia of positive cases [17,124]. It is apparent that the diagnosis of cases is of the utmost importance. However, for both horses and humans, a definitive test relies on the isolation of the bacteria. However, this technique’s ability to determine a positive case is limited by the concentration of bacteria in tissues and biological fluid [12,119]. There are numerous serological tests available for horses, including CFT, ELISA, and agar immunodiffusion, but these assays are restricted by poor sensitivities and are often not specific for Glanders [119,132]. Multiple PCR tests have been attempted, but each is unable to differentiation *B. mallei* from a closely related bacterium, such as *B. pseudomallei* [119,133]. Efforts for human diagnostics have also been unsuccessful, including agglutination, complement fixation, and PCR-based tests [134,135]. The gravity of Glanders warrants urgent diagnostic improvements with modern technology to attempt to impede further large outbreaks.

## 3. Traditional Diagnostic Techniques for Equine Bacterial Diseases

Reliable and accurate diagnostic techniques are vital in the equine industry, not only for the health management of horses but also pre- and post-import or export, pre-breeding screening, and surveillance and prevention of infectious diseases. To ensure consistent and comprehensive reporting of diseases between countries, WOAH and the AAEP assign “gold-standard” diagnostic techniques for crucial diseases [63,96,136] (Table 1). However, many of these deemed “gold-standards” can result in discrepancies amongst different laboratories or users, as certain techniques lack standardized protocols due to the numerous potential methodologies and range of commercially available sample preparation kits [131,132,137]. Furthermore, these diagnostic methods rely on out-of-date techniques, which are time-consuming and require expensive machinery and trained personnel, making them unsuitable for the wider community [138,139,140]. This section outlines current gold-standard techniques assigned to equine bacterial diseases of biosecurity concern and highlights the pitfalls of each.

### 3.1. Bacterial Isolation and Identification Diagnostic Techniques

Bacterial isolation via culturing is one of the most well-known and performed techniques for the detection of equine diseases. Currently, isolation remains the gold-standard for confirmation of Glanders [127,132] and for surveillance of CEM for international trade purposes [110,141]. Whilst this technique is well-established and relatively accessible, several common hindrances persist in culture, such as limited sensitivity, difficulty in differentiation between closely related species, and delayed turnaround of results due to lengthy incubation times [20,142]. Some bacteria, such as *B. mallei* and *S. equi*, require a minimum of 48 to 72 h before growth is visible; this time does not include further biochemical tests required for differentiation, which in turn impedes appropriate responses to disease outbreaks [127,132,139]. Furthermore, it is agreed amongst literature that *T. equigenitalis* is particularly burdensome to isolate, at times requiring over one week to grow [44,141]. Additionally, false-negatives are frequently reported during the isolation process of this bacterium. Researchers suggest this could be a result of numerous elements, such as the bacteria becoming unviable during transport, a low number of bacteria present, overgrowth of unspecific bacteria, or insufficient nutrients in the media [20,141]. Research has been extensively conducted to overcome these matters, for example, incubating *T. equigenitalis* in microaerophilic conditions in conjunction with the use of tryptose chocolate agar [20,143]. However, it has been reported that this does not decrease the length of incubation, with some studies stating growth can still take up to fourteen days [20]. Another study has documented the use of Amies medium with charcoal during transportation periods to increase cell longevity [144]; whilst this offered a promising solution, discrepancies between results continue to occur [20,141].

To overcome the appearance of false-negatives from premature cell death, the United States now requires all *T. equigenitalis* culturing to be performed in a particular certified laboratory. This has resulted in an increased reliability in pathogen detection for the U.S.; however, this policy is not feasible for all regions to implement, particularly those that are resource-poor [20]. Additionally, this solution does not overcome specificity or sensitivity issues, which is a major downfall of this technique. In fact, at times, *T. equigenitalis* colonies are indistinguishable from closely related bacterium [110,145]. This is also the case for *S. equi*, where further biochemical testing must be completed to differentiate from the closely related bacterium *S. equi* subspecies *zooepidemicus* [139]. Yet, culturing is considered the gold-standard for confirmation of a Strangles case in some countries, despite clinical sensitivity being reported at as low as 40% [34,97]. Due to this lack of sensitivity and the lengthy incubation time, quantitative polymerase chain reaction (qPCR) has now replaced the conventional culturing technique as the test of choice in many regions, particularly in the U.S. [96,140]. Replacement of bacteriology testing with molecular diagnostics is an increasing trend observed in equine diagnostics, yet culturing continues to be relied upon for the detection of significant diseases [131].

### 3.2. Serological Diagnostic Techniques

Serological diagnostics encompasses a variety of established techniques such as CFT, ELISA, and IFAT. Each of these methods has been implemented for numerous equine bacterial diseases, taking advantage of the wide range of commercially available tests [146,147,148]. Additionally, sample collection is simple, typically requiring either serum or plasma of a potentially infected animal [147,148]. Whilst serology has been deemed effective in some situations, such as antemortem testing or evaluating seropositivity amongst a large population, these methods rely on the detection of antibody responses and inherently are not suitable in some circumstances [148]. For example, in some cases, the evaluation of disease status using serology may not reflect a current infection, as antibodies post-recovery may still be present [149]. Moreover, antibody titers for multiple diseases may not reach a detectable level until several days or weeks post-exposure [148,150]. Additionally, seroconversion of a disease may be transient, thus relying on specific timing for the test to be conducted. This issue is highlighted using serological testing of CEM, where antibody response in mares reportedly peaks at three weeks post-exposure but starts to decline at six weeks, giving a limited window of opportunity for detection [20,108]. Additionally, it is common practice to require a secondary serum sample to be taken and analyzed 10 to 14 days after initial screening, as antibodies detected in the primary test could be due to past infection or vaccination, thus not reflecting a true positive [147,148]. For example, CFT reportedly can often inaccurately identify horses’ seropositivity, which has resulted in substantial economic losses due to futile international trade and transport restrictions [132,138,151]. In fact, a study reported a false positive rate of roughly 1% in sera analyzed from horses suspected to be infected with Glanders, yet CFT testing is mandatory for the international movement of horses despite numerous reports of contradicting results [132,138]. It has been suggested that CFT should be used in combination with a competitive ELISA assay for a more accurate diagnosis of Glanders [138], but these are both laborious and time-consuming methodologies that further delay advice for action [148,152]. This is particularly harmful to Glanders, where horses must be euthanized upon infection [12,17]. Instead, it is suggested that serological diagnosis should be accompanied with a secondary test such as culturing or molecular diagnostics [132]. These significant disadvantages compared with other available tests calls into question their value and appropriateness as clinical diagnostic methods.

### 3.3. Molecular Diagnostic Techniques

The use of molecular-based methodology has become increasingly favored in diagnostics. This is also true for equine diagnostic laboratories, where techniques such as conventional or quantitative (real-time) PCR are routinely performed [131,153]. Owing to the high sensitivity and specificity, there is strong agreement amongst literature that PCR-based procedures can replace traditional bacteriology, which is often deemed inadequate [131]. Whilst the benefits of molecular diagnostics are compelling, they come with inevitable drawbacks. Firstly, due to the high sensitivity of these assays, the chance of false-positives from contamination or cross-reaction with closely related organisms remains high [16,131,147]. Alternatively, as these techniques are dependent on a gene of interest and thus their sequence, if a particular disease loses this target during mutation, false-negatives can occur [139,147]. Furthermore, stringent purification procedures are required on samples due to a range of PCR inhibitors commonly found in the environment, such as those in feces and mucous [16,131,147]. False positives may also arise in post-amplification visualization using agarose gel electrophoresis due to contamination from operator error via sample spill over, thus requiring specialist trained personnel to perform the entire procedure [32,147].

To overcome contaminate amplification and to enhance PCR applications, qPCR was developed as a real-time alternative [32,131]. Whilst this has been deemed a powerful technique and provided immense progression in disease diagnosis, discrepancies in results continue to occur. Boyle et al. [154] observed this inconsistency when assessing the gold standard qPCR assay for Strangles, which utilizes internal positive control. Here, it was shown that despite returning a positive result using the control, only 2.3% of samples tested negative on initial evaluation; however, 40% of those turned positive after repeating the purification requirements [154]. This example highlights inconsistencies that can occur with molecular diagnostics, which could lead to either lengthy and costly quarantine procedures in the case of a false-positive, or in the case of Glander unwarranted euthanasia, or detrimental outbreaks if a false-negative would occur. Thus, diligence is essential during sample preparation and visualization of results, emphasizing the requirement for qualified personnel for PCR-based diagnostics. Secondly, PCR and qPCR are considered to have a more complex development and preparation in comparison to more traditional techniques, particularly as there is no universal standard protocol [155]. Finally, in comparison to other techniques, such as cultures or biochemical testing, PCR-based methods have high costs associated with equipment set up and maintenance. In fact, for a laboratory to establish an appropriate facility for PCR-based procedures, expected costs are around USD 30,000 and require roughly USD 700 annually for maintenance [156,157]. Nevertheless, for laboratories with appropriate amenities, PCR- or molecular-based diagnostic technology can further advance detection and surveillance protocols, provided current drawbacks are addressed.

## 4. Loop-Mediated Isothermal Amplification (LAMP) for Equine Bacterial Diseases

Continuous advancements in molecular diagnostic technology have aided in the development of rapid isothermal techniques, which can be performed using a single heat source and thus have in-field applicability. Nucleic acid amplification via LAMP is one of the most sought-after of these methods, particularly in equine pathogen diagnostics [38,39,48]. This nucleic acid amplification technique was designed and developed to overcome recurrent drawbacks of most other molecular-based testing, such as the cost, time, and inconsistent results, as outlined above [38]. LAMP employs a simplistic procedure, using four to six primers targeting selective regions of a sequence on the sense strand with additional complementary regions on the anti-sense strand [158]. As the name suggests, this technique is performed at a constant temperature of around 65 °C, which is suitable for both the polymerase and primers, allowing for strand displacement and amplification to occur simultaneously [38]. Furthermore, LAMP has been noted to have comparable sensitivity and specificity to both qPCR and nested PCR due to the use of multiple primers at relatively high concentrations [38,158]. LAMP can also tolerate typical inhibitors that impact other amplification methods and thus can be used for a variety of samples, such as blood, tissues, and feces, without the need for lengthy purification methods [39,159]. Perhaps one of the most enticing features of this technique is the rapidness of amplification, where results can be confirmed within an hour or often in less than 20 min [45,158]. The resulting output is simple and can be provided as either an endpoint application or in real-time with straightforward monitoring techniques, reducing the need for expert personnel and providing a vast range of applicable users. Common monitoring methods currently involve measurements of either fluorescence signals, using fluorescent chelation reagents incorporated into the mastermix, or turbidity, which utilizes the magnesium pyrophosphate that is produced as a by-product during DNA synthesis. Both methods allow for visual inspection in real time. Real-time detection methodologies have been a focal point for research to provide an array of in-field applications [39], which is discussed in the section below.

Whilst LAMP has been described as revolutionary to microbiology, some of its features can have adverse effects. For example, the use of multiple primers at a high concentration relative to other amplification techniques can result in primer hybridization or non-target amplification [49,160]. Yet, numerous advancements in recent years have aided in overcoming these issues and even increased certain elements, such as time to results, sensitivity, and specificity [49]; these are further detailed in the next section. Nevertheless, LAMP has been demonstrated to be a reliable molecular diagnostic method that addresses and overcomes common difficulties seen in traditional techniques [32,158].

### Current Applications of LAMP for Equine Bacterial Disease Diagnostics and Surveillance

As equine diagnostics and surveillance procedures continue to progress, in-field techniques have become favorable; thus, research and development have largely focused on LAMP. Numerous assays have been developed for diagnostics and surveillance of equine viral [16] and parasitic diseases [47,48]. Moreover, extensive attention has been paid on bacterial diseases, particularly those of biosecurity concern (Table 2).

Kinoshita et al. [161] developed a LAMP assay for the specific detection of CEM targeting the 23S rRNA gene. The assay’s analytical sensitivity (interchangeably noted as the limit of detection across studies) regarding the least amount of target DNA present in the sample to yield a positive result was 24.8 copies of DNA per reaction of both cultured strains and spiked horse genital swabs. When comparing this assay to a semi-nested PCR test, Japan’s official test for case confirmation [116], it was found to have a similar sensitivity. Yet, the authors state the usefulness of the LAMP assay is greater as the time to result, which is the required length of time for assay completion, is under an hour when using a turbidimeter for real-time detection, compared to several hours for the semi-nested PCR assay [161]. The same group later compared the LAMP assay to six PCR-based methods, including the semi-nested PCR assay, a real-time PCR assay, and four conventional PCR assays [165]. However, this showed conflicting results, with both the semi-nested and real-time PCR assay reporting 78% for clinical sensitivities, being the accuracy of correctly identifying positive and negative results, whilst the LAMP assay had a lower sensitivity of 71%. Additionally, the real-time PCR assay’s limit of detection was much lower than LAMP, which was 1.2 copies per reaction compared to roughly 25 copies per reaction, respectively. Despite this, the authors agreed with their previous statement that LAMP would be of greater use in clinical settings, due to the rapidness and lower cost. Moreover, the authors note clinical samples for diagnosis of CEM require genital swabs which can frequently be contaminated with feces or urine, and as previously discussed, can inhibit PCR assays as opposed to LAMP. Furthermore, the LAMP assay could be deployed in-field whereas each six of the PCR-based methods are limited to laboratory use only.

Due to the critical nature of Glanders and the detrimental ramifications if an outbreak occurs, there have been multiple LAMP assays designed for rapid detection, each taking advantage of real-time turbidity monitoring [12,46,162]. In 2016, Mirzai et al. [162] developed an assay detecting the integrase gene cluster of Glanders, which can be performed in less than 60 min. However, the assay’s limit of detection was relatively high at 22 ng/µL. At the time, the authors reported the integrase gene was highly specific to *B. mallei* strains, and as such, there was no evidence of cross-reactivity. However, further research revealed this gene is also present in thirteen strains of *B. pseudomallei*, making it unsuitable for definitive detection [12]. As a result, Pal et al. [46] 2018 produced an assay to target the *BMA10229_0375* gene of Glanders. This assay showed a greater limit of detection, identifying 1 pg per reaction within an hour when using culture strains and 5.5 × 10^3^ colony-forming units (CFU) per ml in artificially spiked human blood. Saxena et al. [12] then, in 2019, developed an assay targeting the gene *fliP*-IS*40J*A, with a considerably lower limit of detection of 0.25 pg per reaction of genomic DNA and 2.1 × 10^3^ CFU/mL also in artificially spiked blood. This assay was able to be performed in roughly 52 min and did not detect either closely related pathogens or bacteria frequently isolated from horses. Together, these assays demonstrate the ability of LAMP methodologies to advance in just a few years.

Further demonstrating the analytical sensitivity of LAMP, Kinoshita et al. [44] published an assay detecting *R. equi* with a limit of detection of just 10 CFU/mL in tracheal washes of horses’ with lower respiratory infection. The assay, which targeted the *vapA* gene, had equivalent specificity of roughly 94% when compared to a qPCR and semi-nested PCR assay. However, qPCR yielded a greater clinical sensitivity to that of LAMP, at 97.1% and 94.1%, respectively. Despite this, the authors again stated a LAMP assay does not require the expensive machinery that PCR-based methods require. Moreover, the LAMP assay was able to be performed in under 25 min, compared to over an hour for amplification via qPCR. In agreement with Nemoto et al. [43], Parida et al. [155], and Notomi et al. [158], the authors concluded that LAMP could be deployed for laboratories that are less well-equipped or are resource-poor, highlighting the versatility this technology possesses [44].

To date, there are two developed LAMP assays that rapidly detect Strangles cases, both with the ability to be utilized in routine surveillance programs [45,163]. In 2012, Hobo et al. [163] developed an assay utilizing the *S. equi*-specific gene *seM* as the target gene and monitored results in real-time using a turbidimeter. The optimized assay was compared to a semi-nested PCR test, which resulted in equivalent clinical sensitivity (not reported) and specificity (100%). However, the LAMP assay had a higher limit of detection of 0.1 CFU/mL compared to the PCR-based test, 0.01 CFU/mL. Yet, the authors noted this level of analytical sensitivity is sufficient for clinical appraisal of cases. As seen in previously discussed literature, the LAMP assay was significantly quicker compared to semi-nested PCR, taking only roughly one-third of the time when using tracheal washes.

Another group, Boyle et al. [154], compared a previously reported Strangles LAMP assay [164] detecting the *S. equi*-specific gene, *eqbE*, to a real-time PCR assay that detects the *seeI* gene. However, this assay was not as analytically sensitive as the assay developed by Hobo et al. [163], with a limit of detection of 1 CFU/mL. While the real-time PCR assay had a greater clinical sensitivity (83%) compared to the LAMP assay (77%), the specificity of LAMP was preferable, 65% and 78%, respectively. Again, the authors also argue the LAMP assay’s time to completion (~30 min) demonstrates superior practicality over real-time PCR (~70 min) and culturing (~3 days) [154].

More recently, the same group has applied a previously developed in-field detection method that uses a microfluidic device that can be performed with a smartphone [45,166], which is discussed further in the section below. This device’s performance was compared to both a benchtop version of the LAMP assay and a triplex qPCR assay that had become commercially available for point-of-care use [139]. Interestingly, the qPCR assay only detected twelve of sixty-seven samples being positive, despite an internal control identifying sixty-three, emphasizing the inconsistencies that can arise from PCR-based methods. Additionally, the research group found that the LAMP assay using either the microfluidic device or a benchtop assay had greater clinical sensitivity (100% and 92%, respectively) compared to the qPCR assay (89%). However, it should be noted that qPCR was more specific at 84% in comparison to 62% and 71% for the microfluidic device and benchtop assay, respectively. The limit of detection was 1 CFU/mL for both LAMP assays, yet both could be performed within 15 min. Despite some drawbacks, the microfluidic device offers exciting insight for further development of real-time and in-field monitoring methodologies for LAMP [45].

## 5. Current Advancements in LAMP Technology

The advantages of LAMP over traditional detection and surveillance techniques are indisputable, particularly assay time and cost [39,155]. However, for LAMP to replace embedded traditional techniques in the equine industry, these assays must exceed diagnostic criteria. As such, assays should be designed and developed to surpass the clinical and analytical sensitivities of other techniques rather than be comparable. Additionally, result monitoring methods should aim to use minimal equipment to provide complete in-field capabilities. Thus, further research and advancements of this methodology are continuously growing, focusing on increasing assay kinetics and capabilities and developing accessible monitoring protocols and technology [32]. This section briefly outlines research involving chemical additives and expansion of endpoint and real-time detection, all of which can be easily implemented to strengthen current and future LAMP assays for equine bacterial diseases.

### 5.1. Chemical Additives for the Advancement of LAMP Assay Capabilities

Chemical additives have long been researched and utilized in nucleic acid amplification assays, in particular PCR, and have thus provided a backbone for the utilization of LAMP techniques [49]. The overarching aim for the implementation of additives focuses on increasing sensitivity, specificity, and limit of detection and decreasing time to result and non-specific amplification whilst stabilizing the robustness of the assay [49,167,168]. An extensive review of current additives used for nucleic acid amplification has been previously published by Özay and McCalla [49]. However, this section will focus on specific additives that have been applied to LAMP and their differing successes.

#### 5.1.1. Enhancement of LAMP Assay Kinetics and Proficiency

Whilst the impressive rapidity of LAMP has gained much attention, numerous reports show the limit of detection is equivalent or even subpar to that of PCR-based assays [44,163,165]. This has somewhat constrained the adoption of LAMP as a customary technique for diagnosis and surveillance. However, researchers agree this isothermal technique should not be overlooked and can revolutionize modern-day molecular diagnostics [39,45]. Whilst a LAMP reaction mixture can have variable compositions between companies, typically, the reaction mixture will include deoxyribonucleotide triphosphates (dNTPs), betaine, potassium chloride (KCl), ammonium sulfate ((NH_4_)_2_SO_4_), magnesium sulfate (MgSO_4_), and the *Bst* DNA polymerase [38,158]. These components provide the foundation for nucleic acid amplification and reaction mixture stability for a viable commercial product. More recently, however, a relatively broad range of research on assay additives has been conducted to enhance assay kinetics and proficiency [49]. Furthermore, LAMP provides accessible point-of-care (POC) operation; as such, research into stabilizing reagents for long-term storage is expansive to ensure stability and reliability when performing diagnostics in the field.

Previously, it had been determined that the addition of L-proline can increase salinity tolerance, thus assisting in sustaining DNA polymerase activity and lowering the melting temperature of DNA [169,170]. Nyan et al. [171] created a thermostable LAMP reaction mixture buffer using L-proline, which allowed for reagents to be stored at room temperature for six months whilst retaining the activity of the reaction mixture. Additionally, despite substandard storage of reagents and preparation conditions, both the clinical and analytical sensitivity did not diminish, highlighting the use for POC applications as the reaction mixture displays considerable flexibility with the addition of L-proline. The disaccharide trehalose is also a reliable thermal stabilizer for multiple enzymes and is frequently used in lyophilized LAMP reagents to aid in POC use [49]. Impressively, Curtis et al. [172] found the use of 5% (*w*/*v*) trehalose can stabilize LAMP reagents in lyophilized form for 27 days when stored at 30 °C. Chen and Ching [173] agreed with this study when they observed reagents remained stable for 28 days at room temperature with 5% (*w*/*v*) trehalose, as well as storage at 37 °C for 2 days. The study noted a slight decline in the limit of detection compared to reagents stored at 4 °C. Nevertheless, the authors note these results suggest the addition of trehalose can replace the need for dry ice during transportation, which can substantially lower the cost of freightage and, therefore, the cost of production. However, it is clear optimization of the trehalose concentration is required as Carter et al. [174] noted a modest decrease in polymerase activity after 18 days at room temperature, although it should be noted this study used a 10% (*w*/*v*) concentration of trehalose rather than a 5% (*w*/*v*) concentration used in the aforementioned studies. Interestingly, the same study found reagent stability with trehalose increased when omitting SYBR green dye from the lyophilized mix. Furthermore, Wan et al. [175] combined 5% trehalose with 1.5% glycine in the lyophilized reagents; however, this only stabilized reagents for 3 days at temperatures between 24 °C and 30 °C. Contrary to previous studies, these authors observed trehalose alone had “adverse effects” on the assay; however, they did not report what these effects were. It has been theorized that this could be a subsequent effect of trehalose reaching a solubility limit due to the potential precipitation of trehalose, limiting the analytical sensitivity [176]. Nevertheless, studies exploring the use of these chemicals reinforces the important durability and sustainability in long-term and suboptimal storage of LAMP reagents, a critical requirement for POC diagnostics and surveillance [174,177].

Owing to its robust nature and tolerance of typical amplification inhibitors, LAMP can theoretically employ similar chemical enhancers that have been trialed for other nucleic acid amplification assays. Indeed, the addition of such enhancers has been shown to give greater responses, such as lowering the limit of detection and time to results [39,159]. However, it should be noted this is not always the case, as literature often reports conflicting findings. For example, betaine has been advocated as a LAMP additive to increase sensitivity and specificity and is, in fact, a typical component in many DNA amplification reagents [38,49]. Early PCR-based studies suggested during amplification, betaine decreases the melting temperature of double-stranded DNA, thus increasing the limit of detection [178]. Yeh et al. [179] applied this information to observe the effect in LAMP assays. Through a concentration gradient, the study determined 0.8 M of betaine increased assay kinetics; however, higher or lower concentrations had the opposite effect. This was also observed in a study conducted by Ma et al. [180], where higher concentrations of betaine resulted in a stronger inhibitory effect of fluorescence signal. Contrarily, a study conducted prior by Zhou et al. [181] found the same high concentration of betaine had no significant effect on signal output compared to their control, which had no betaine. It is hypothesized the inconsistent reporting of betaine functionality amongst nucleic acid amplification techniques could be a result of different primer and DNA sequences among studies [182]. As the gene target used by Zhou et al. [181] was AT-rich, this could explain the underwhelming results.

Despite inconsistent results of some additives, multiple studies conducting a LAMP assay targeting SARS-CoV-2 have found exceptional results when adding guanidine hydrochloride (GuHCl) [183,184,185]. Each study has found a significant increase in sensitivity and a roughly 40% decrease in the time to result. Zhang et al. [183] were the first to report on this phenomenon where they found GuHCl consistently raised the clinical sensitivity by around two-fold, with 50 copies of target RNA per reaction increasing from a 30% positive rate to 70% and 100 copies per reaction increasing from 50% to above 90%. Due to this impressive result, GuHCl is now a standard component in the New England BioLabs (Massachusetts, United States) LAMP detection protocol for SARS-CoV-2 [184]. The mechanism of action of GuHCl remains unknown, but it is speculated it may strengthen the base pairing of primers to the target DNA or RNA sequence. Whilst this assay was originally conducted using RNA, which requires an additional enzyme for reverse transcription, the study also tested the use of GuHCl on DNA and found equivalent results (unpublished data) [183]. This suggests the mechanism is not sequence-dependent and does not interact with polymerase activity [183]. These results provide a new foundation for enhancing assay kinetics using GuHCl and should be explored further to cement LAMP as a pivotal tool for diagnostics.

#### 5.1.2. Reduction in Non-Specific Amplification

Non-specific amplification is a common complication in nucleic acid amplification techniques, typically arising due to primer-primer interaction resulting in primer-dimer formation or partial hybridization of a primer with non-target DNA sequences [167,186]. This is especially notable in LAMP assays due to the requirement of additional primers at high concentrations. Additionally, the relatively low amplification temperature LAMP requires can initiate secondary structure formation of DNA [49,186]. However, multiple chemical additives have been explored to suppress or completely inhibit this outcome [49]. Pullulan, a polysaccharide polymer, has been suggested as an additive to partially suppress primer-dimer formation that results in non-specific amplification, following its success in other nucleic acid amplification techniques such as cross primer amplification (CPA) and rolling circle amplification (RCA) [187,188]. When tested with LAMP, Gao et al. [189] found pullulan could reduce non-specific amplification whilst not impacting assay performance. The study proposed pullulan could potentially stabilize primers to decrease primer-dimer formation, inhibiting non-specific amplification. As a polymer, it is possible pullulan could form micelles with DNA and thus encapsulate primers to prevent hybridization between primers in the absence of target DNA, a theory that was presented by Liu et al. [188] when testing pullulan as a stabilizer in RCA. It is apparent pullulan can be a potentially useful additive for the suppression of non-specific amplification in LAMP assays [189].

The incorporation of dimethylsulfoxide (DMSO) has previously been shown to enhance the limit of detection in PCR-based techniques by hindering the formation of secondary structures and has since been advocated as one of the most successful additives for such assays [190,191]. Wang et al. [192] successfully applied this theory to a LAMP assay aiming to improve analytical sensitivity and specificity. While the study found high concentrations of 10% (*v*/*v*) DMSO may inhibit enzyme activity, they determined a concentration of 7.5% (*v*/*v*) can elevate the amplification rate of targets with low concentrations. Garrido-Maestu et al. [193] consolidated these findings when replacing betaine with DMSO and also found that 7.5% (*v*/*v*) was an optimal concentration to increase specificity. Furthermore, while this study found no substantial increase in time to result of the assay, the same group later found an assay timing decreased with the additive when developing a different LAMP assay [194]. Later, a study by Shahbazi et al. [195] used DMSO in conjunction with betaine to decrease non-specific amplification and, subsequentially, increased the specificity of their LAMP assay. The study determined this combination had decreased false positives by utilizing DMSO as an enhancer to betaine’s functionality rather than changing the polymerase and primer kinetics themselves. However, a recent study conducted by Jang and Kim [167] agreed with earlier reports that DMSO can have adverse effects on enzyme activity at high concentrations of 7.5% (*v*/*v*).

In addition, the group evaluated various other chemical additives, such as formamide, Tween 20, and bovine serum albumin, which resulted in either no significant suppression or an increase in non-specific amplification. Yet, the study showed the successful use of tetramethylammonium chloride (TMAC) as an assay enhancer [196]. This study found efficacious suppression at 20 mM (*v*/*v*) of TMAC with no effect on the LAMP reaction; however, higher concentrations (40–60 mM) resulted in a minor but insignificant timing delay [167]. Given the results from this study, TMAC appears as a promising suppressive reagent for non-specific amplification in LAMP assays; however, future investigations are required. Despite the predisposition for non-specific amplification in LAMP, these above studies show that both pullulan and TMAC can minimize this effect or even completely inhibit it. Furthermore, the flexibility of supplementary assay enhancers that LAMP can tolerate, in comparison to other nucleic acid amplification techniques, warrants further research into additional chemicals that may facilitate greater results.

### 5.2. Advancements in LAMP Monitoring Techniques and Technology

Research and development of field deployable accessible monitoring techniques has gained copious attention in recent years. The prominent techniques to be exploited are simple, accurate, and stable for use in remote or resource-poor communities [39]. The ability to amplify target DNA via LAMP still requires a detectable readout, commonly via a machine for fluoresce detection in laboratory conditions; however, other detection methods have been adapted for a one-use, cheaper readout system. Recently, an abundance of research has focused on newer technology, taking advantage of previously established nucleic acid monitoring technology and applying it to LAMP, for example, lateral flow dipsticks (LFD) and biochips, “lab-on-a-chip” (LOC) [197,198]. This section will outline real-time turbidity and fluorescence detection, both commonly used techniques, as well as newer technology that can transform LAMP monitoring applications.

#### 5.2.1. Conventional Monitoring Procedures Commonly Utilized in LAMP

Currently, the measurement of turbidity and fluorescence signals are the standard monitoring techniques for LAMP. Both techniques can be performed as either endpoint or in real-time, without the need for specialized methodology or expensive machinery [39,46,154]. Turbidity monitoring involves the evaluation of pyrophosphate precipitate, which is generated in large quantities as a by-product of DNA synthesis from dNTPs. During the reaction and hybridization of DNA strands, pyrophosphate ions are released and bind to magnesium ions, thus forming a white precipitate that can be observed by the naked eye [39]. As turbidity is a naturally occurring process during amplification, due to the presence of additional magnesium at a higher concentration compared to a traditional LAMP reaction mixture, extra running costs are only required for real-time monitoring where either a turbidimeter or spectrophotometer is utilized to detect luminescence from light emitting diodes [199,200]. Additionally, turbidity monitoring, either endpoint or real-time, is considered the most simplistic method of analysis and does not require specialized indicators or probes. The risk of amplicon contamination is also removed as tubes remain closed for the entire process after assay preparation for visualization of results [39]. However, as with many naked-eye detection techniques, there is a chance of result misjudgment of results due to visual interpretation errors. It should also be noted magnesium pyrophosphate particles may potentially redissolve, resulting in false positives or relatively lower sensitivity, although this phenomenon is considered rare [201]. Thus, to overcome sensitivity issues, the detection of optical signal changes through fluorescent dyes was developed, although at a higher running cost [39]. Intercalating dyes in the reaction immediately bind to double-stranded DNA products upon synthesis and fluoresce, which can be monitored in real-time and is, in fact, reportedly up to 50% faster in detecting a positive sample compared to turbidity [199]. Whilst both techniques allow for direct indication of results, the additional machinery that is required, such as a real-time fluorescence detection device, may limit POC use. To resolve this, methodologies combining fluorescence and metal indicators could prove to be more suitable for in-field use. These methods provide results as endpoint detection; however, visualization only requires a light source, whether using ultraviolet light or a well-lit room [39,200].

**Figure 1:**
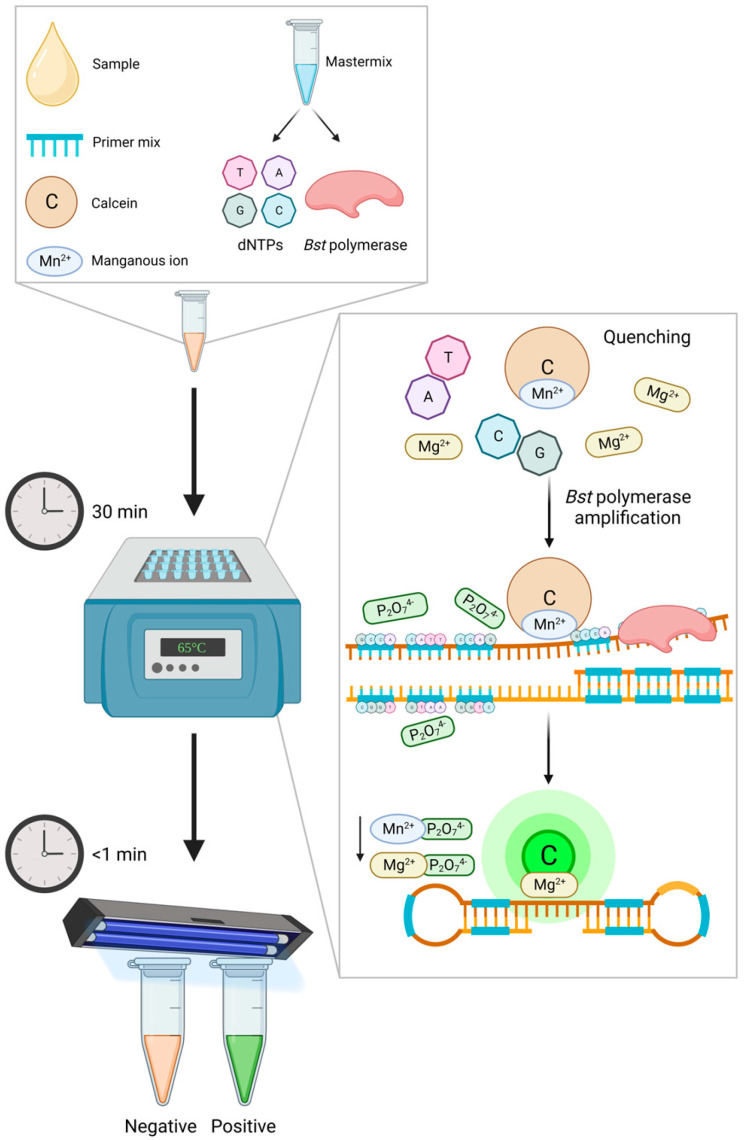
Visualization of LAMP results using calcein as a fluorescence metal indicator. Calcein and manganous ions (Mn^2+^) are added with reagents in a reaction tube, which is heated using a heat block for 30 min at 60–65 °C. Initially, Mn2+ binds with the calcein to quench fluorescence, resulting in the reaction mixture appearing orange. A *Bst* polymerase aids in the LAMP procedure and DNA synthesis, during which pyrophosphate ions (P_2_O_7_^4−^) are produced as a by-product from dNTPs amplification. The P_2_O_7_^4−^ will preferentially bind to Mn^2+^ and release the calcein, creating a fluorescent signal. Free calcein is then able to bind with residual magnesium ions (Mg^2+^), which are in the master mix, resulting in a strong, UV or naked-eye visible fluorescent glow. Created with Biorender.com.

One such example is the use of the fluorescein complex calcein and its natural quencher manganous, which are both directly added to the mixture during assay preparation (Figure 1). The manganous ions bind to calcein prior to amplification and suppress calcein’s fluorescence, giving the non-amplified reaction an orange tone. During DNA synthesis, pyrophosphate ions are produced as a by-product and strongly bind to the manganous ions, thus releasing the calcein and, in turn, creating a green fluorescence [202]. This signal is enhanced when released calcein binds with the assay’s residual magnesium ions, and this color change can be visualized using the naked eye. As the release of calcein is proportional to the number of pyrophosphate ions being generated during DNA synthesis, the fluorescence intensity indicates a higher concentration of target DNA [200]. However, as mentioned, caution should be taken when using naked-eye visualization, as one’s perception of color can differ from another, suggesting the possible need for trained personnel [203].

#### 5.2.2. Lateral Flow Device

**Figure 2:**
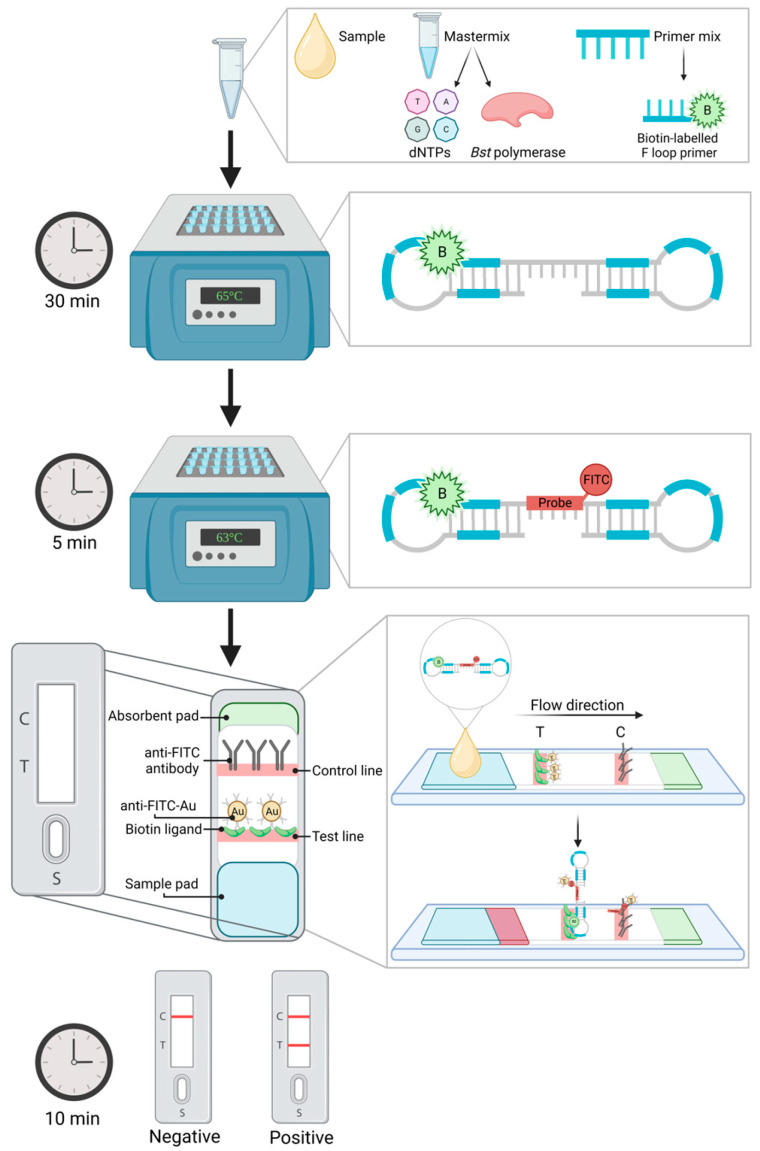
Schematic diagram of LAMP coupled with a lateral flow device (LFD) utilizing a biotin-labeled forward loop primer. The LFD cassette contains a sample pad for sample application, a “test” (T) and “control” (C) line separated with a nitrocellulose membrane, and an absorbent pad for overflow. Samples undergo the LAMP procedure on a heat block for 30 min at around 65 °C and produce a biotin-labeled amplicon, which is then hybridized to a fluorescein isothiocyanate (FITC) probe for 5 min at 63 °C. The product, diluted in wash buffer, is injected into the LFD sample window (S) and flows through to the T line. Biotin-labeled amplicons are captured at the T line, and gold-labeled anti-FITC (anti-FITC-Au) antibodies bind to the FITC probe on the amplicon, which produces a dark band. Anti-FITC-Au bound FITC probes, which are not captured using biotin, continue to the C line and produce a dark band. Results are considered positive if lines appear at both C and T, whereas negative results are indicated using a single line at C. Created with Biorender.com.

Lateral flow dipsticks (LFD), an immunochromatographic technique, have frequently been adapted for the visualization of results in multiple nucleic acid amplification procedures [39,204]. These small portable cassettes act similarly to enzyme-linked immunosorbent assay (ELISA) in that they employ specific antibody capturing and secondary labeling [203,205,206]. The procedure of LAMP combined with LFD is outlined in Figure 2. Briefly, a LAMP primer, typically either one of the inner or loop primers, is labeled with biotin, which will bind with the synthesized target DNA during amplification. After the LAMP procedure, the biotin-labeled amplicon is hybridized with a fluorescein isothiocyanate (FITC) probe for typically 5 min in a separate tube [197,204]. This biotin and FITC labeled product is then diluted in a wash buffer and injected onto the sample pad in the cassette and flows across the cassette towards the absorbent pad, passing across a “test” and “control” line. At the test line, biotin ligands capture the biotin-labeled LAMP product, whilst gold-labeled anti-FITC antibodies bind to the hybridized FITC to form a triple complex. Once “trapped” and bound, the gold-labeled anti-FITC antibodies develop into a dark band on the test line [206,207]. The remaining FITC probes that have not been captured using biotin will form a double complex with the gold-labeled anti-FITC and migrate to the control line, again producing a dark band to indicate a successful test. Thus, a positive sample is observed by two visible lines, one at both the test and control windows [203,204,207].

An early study exhibited the successful combination of LAMP with LFD when utilizing FITC-labeled DNA probes. This study reported a total time from sample collection to visualization of results within 50 min, with a limit of detection at 1 picogram of DNA per ml [206]. Further studies have reported varying detection limits; nevertheless, each report agreed that LFD is a superior detection method to SYBR Green I colorimetric methods [197,207,208]. In fact, Diribe et al. [204] reported their LFD had a limit of detection of 10 gene copies (equivalent to 0.0052 femtograms per microliter) within 30 min of amplification. The authors noted that while real-time detection using turbidity or fluorescence was more clinically sensitive over the LFD, the difference was minimal at 97% and 95% sensitive, respectively. Furthermore, Diribe et al. [204] suggested the required primer modification for LFD does not have adverse effects on LAMP reactions. The same group developed another assay targeting *Pseudomonas aeruginosa* from equine genital swabs, again utilizing both FITC probes and biotin-labeled primers [203]. For these assays, LFD monitoring was slightly more clinically sensitive than real-time fluorescence, at 88.5% and 86.8%, respectively. Whilst both analytical and clinical sensitivity is comparable to real-time monitoring, LFD provides an extra level of specificity through the requirement of both biotin and FITC detection [204]. Furthermore, it is agreed amongst literature that LFD allows for unambiguous results with clear indication through the presence or absence of lines, compared to the operator interpretation of turbidity or fluorescence color detection [203,204,206]. Whilst the development of such methods can be time-consuming and costly, the application and rollout to a wide range of equine facilities is straightforward and rapid, with no requirement for specialized equipment. Thus, LFD is quickly proving to be a promising candidate for equine POC diagnostic testing [39].

#### 5.2.3. Microfluidic Devices Coupled with Biochemical Chips

**Figure 3:**
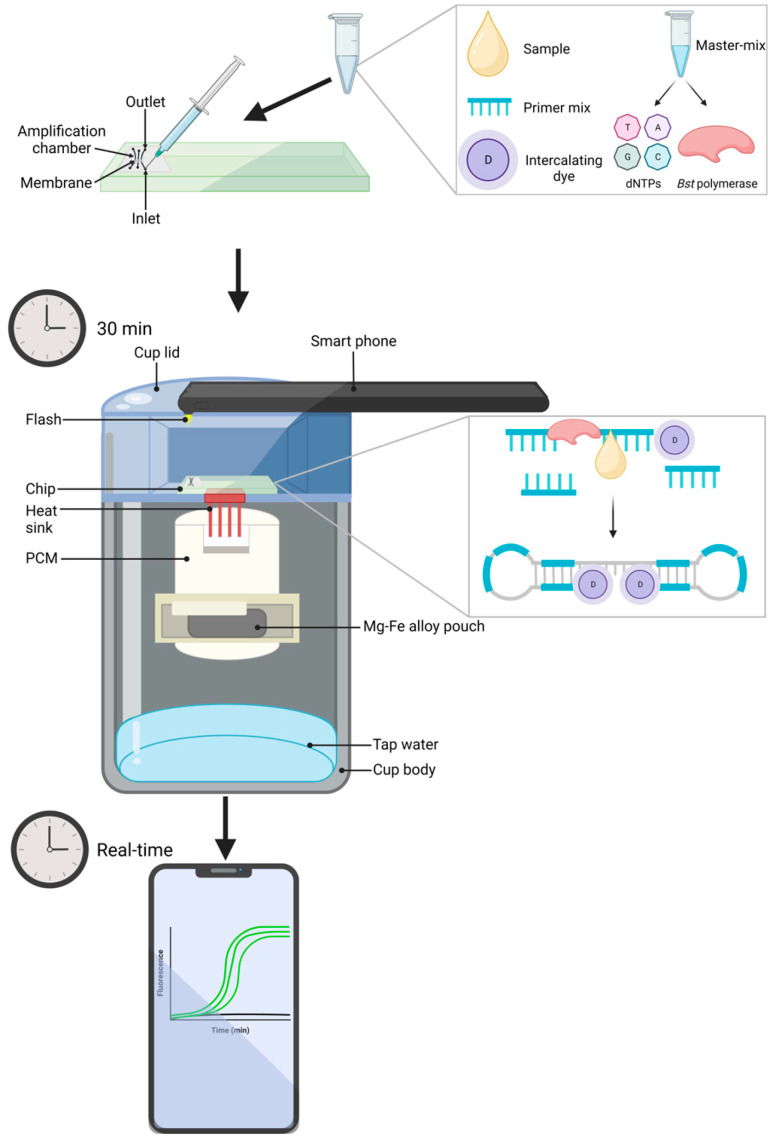
Representation of simultaneous amplification through LAMP and visualization of results through fluorescence detection coupling a biochip with a smartphone and “Smart Cup”. The microfluidic chip contains several inlet ports for the assessment of multiple samples in one assay, outlet ports, as well as a membrane to capture nucleic acid, and an amplification chamber where heating occurs. A sample is mixed with a binding reagent to assist with dNTPs adsorption and capture onto the membrane and is injected into the inlet port. The membrane is then washed with a high-salt ethanol-based buffer that is injected into the inlet whilst the nucleic acid remains captured. After airdrying for around 30 s, the LAMP reagents, including the primers, are injected. Both the inlet and outlet are sealed tightly with tape. Prior to insertion of the chip, water (~7 mL) is added to the smart cup, which activates the heating of the Mg-Fe alloy pouch, and the phase-change material (PCM) maintains the temperature, in turn heating the heat sink. Once heated to the appropriate temperature (~60–65 °C), the chip is inserted, and the LAMP procedure occurs. A smartphone’s flashlight is used to excite fluorescence emission. These fluorescence signals are captured using the charged couple device (CCD) camera and can be visualized on the smartphone in real time. Created with Biorender.com.

Perhaps one of the largest breakthroughs in LAMP monitoring research is the use of microfluidic devices coupled with biochips, or lab-on-a-chip (LOC), systems. These combined methods have gained copious attention for POC applications, as LOCs and supporting instruments, if required, are portable and are performed in real time [39]. Additionally, due to a relatively hands-off approach, assays can be conducted using non-trained personnel, allowing for accessibility in a range of communities [166,208]. In fact, Liao et al. [166] have developed a successful electricity-independent device, termed “Smart Cup”, using simplistic materials, such as a Thermos cup and a smartphone, that integrates a LOC (Figure 3). The LOC utilizes microfluidic networks, where samples are injected into individual inlet valves, followed by sample lysis, and then the soluble fraction is filtered through the membrane, which captures any nucleic acid present in the sample. The membrane is then washed, typically with a 50:50 ratio of water and ethanol, and the required LAMP reagents are injected, following which the inlet and outlet ports are sealed with tape. The Smart Cup is then heated to the appropriate temperature through the addition of water, which activates a Mg-Fe alloy pouch. After reaching a stable optimal temperature, the LOC is placed onto the stage where heat is maintained through phase-change material (PCM) and a heat sink, which delivers the heat to the chip. As the LAMP procedure begins, a smartphone’s flashlight is turned on to excite fluorescence emission, which is recorded using the phone’s charge-coupled device (CCD) camera [166,208]. Alternatively, the LOC can be preloaded with LAMP reaction mixture and primers and sealed using paraffin, which will melt once the temperature reaches roughly 60 °C and subsequently release the reagents and combine with the DNA template [208]. Images or video recordings of the fluorescence signals that are captured using the CCD camera can be analyzed in real-time or post-amplification. When testing their developed device, Liao et al. [166] observed identical results compared to a benchtop LAMP detection machine, both having a limit of detection at 100 copies. The authors also note the Thermos cup could be replaced with a Styrofoam cup as a cost-saving option; it should also be noted the Mg-Fe allow pouch only costs a mere US$ 0.15 each, although they are single use only. This Smart Cup could very well transform POC diagnostics. Boyle et al. [45] utilized this technology to test a LAMP assay detecting equine Strangles. With the Smart Cup, the assay could be performed in less than 15 min, with a 100% clinical sensitivity and limit of detection of 1 CFU. While the specificity of the LAMP assay was lower than that of commercially available PCR assay, 62% compared to 84% respectively, the greater clinical sensitivity and lower limit of detection of the microfluidic device can aid in detection of Strangles in convalescent horses where a low bacterial load is expected. Together these papers demonstrate and highlight LAMP coupled with microfluidic devices and LOCs as a powerful tool that should be thoroughly researched as an accessible POC technique for equine bacterial diseases [45,166].

## 6. Current and Future Priorities for Equine Bacterial Disease Diagnosis

The prominence and damaging effects of previous equine bacterial disease outbreaks demonstrate the urgency for rapid and precise diagnostic techniques whilst maintaining a simplistic and accessible method [3]. The robust nature and ease of use of LAMP, coupled with continuous advancements, gives little doubt as to why this technology is being rapidly developed in research for equine disease diagnosis and surveillance [16]. Yet, despite numerous assays being designed and optimized for equine bacterial diseases of biosecurity importance, LAMP has failed to replace gold-standard techniques thus far. This could derive from a gap in communication between researchers and those who perform diagnostic procedures. Further advocation regarding the advantages of LAMP, such as the cost and time of such assays, could potentially help in overcoming this. Furthermore, as LAMP is designed to have simplistic and flexible methodologies thus not requiring trained personnel, there should be more endorsement for those who directly work with horses every day, for example, farmers and stud owners, to self-manage surveillance using these assays. For such situations, the incorporation of either cost-effective LFDs or LOCs, such as the Smart Cup [166], for disease detection would be ideal. For example, routine screening of equine disease status for *R. equi* is imperative, and whilst a LAMP assay has been developed to detect this disease in under 25 min [44], qPCR remains the customary diagnostic method [63]. This means the sample must be transported to an accredited laboratory and undergo lengthy purification methods before being tested, causing a delay between notification of a suspected case and confirmation [44]. However, if the previously developed LAMP assay were to be optimized using a simple field-deployable LOC or microfluidic device, horse owners could potentially be able to perform testing themselves, thus providing immediate results for appropriate action. Yet, as the *R. equi* LAMP authors noted, despite having equivalent specificity to PCR-based methods, the qPCR had a greater clinical sensitivity. However, the addition of an additive such as GuHCl could improve assay stability and kinetics, as seen in other LAMP assays [183]. These slight modifications could aid in promoting the *R. equi* LAMP assay to become a common routine surveillance method across equine farms. The same, or similar, adjustments could be made to the LAMP assay developed by Kinoshita et al. [161] to detect CEM to increase the clinical sensitivity. However, as sample collection for this disease can be invasive and thus requires a trained professional, it is likely further development for a simplistic result output would be unnecessary. Similarly, modification to simplify the LAMP assay to detect Glanders developed by Saxena et al. [12] would also be inessential as this highly classified bacterial disease requires strict biosafety laboratories for any form of testing. Whilst it is evident that LAMP could become a breakthrough technique for the equine industry, continuous development and optimization do need to occur. Additionally, bridging the gap of communication between researchers and diagnosticians regarding LAMP assay implementation is essential to advance current diagnostic and surveillance techniques, and continue to protect the equine industry.

## 7. Conclusions

The equine industry remains a vital entity worldwide that contributes to various communities and cultures and substantially provides economic growth. With the ever-increasing movement and trade of horses, and thus the potential for disease transmission, it is essential to continually advance biosecurity measures. Current diagnostic and surveillance techniques are proving to be unreliable or do not serve the wider community due to inaccessibility of expensive machinery. Additionally, the vast majority of current gold-standard detection methods are time-consuming and thus do not allow for immediate action in cases of outbreaks. LAMP, however, has proven to be a sound molecular technique that overcomes most pitfalls associated with other nucleic acid amplification techniques. Further research into improving these LAMP assays has shown promising results that can strengthen this method beyond current capabilities, such as additional experimentation of additives that have been trialed with other nucleic acid amplification techniques. The promising results of the incorporation of GuHCl for increased analytical sensitivity and assay stability and TMAC for the inhibition of non-specific amplification have highlighted the advancement additives can have on LAMP assays. However, as studies for enhancing these assays in equine medicine are limited, further investigation on additive effects for disease detection and surveillance using LAMP is heavily recommended as it is apparent that additive improvement is assay specific. Furthermore, the use of simple field-deployable amplification and detection techniques, such as LFD or LOC technology, may just revolutionize LAMP technology. There is an apparent need for rapid results for the implementation of control measures to prevent detrimental spread and outbreaks throughout the equine industry, and thus, it is suggested that the development of LAMP methodologies should be a focal point of research in equine medicine.

## Figures and Tables

**Table 1:** Assigned gold-standard diagnostic techniques and other common detection methods for equine bacterial diseases of biosecurity concern.

Causative Agent	Disease	Gold Standard Technique * Common Detection Method	Reference
*Rhodococcus equi*	Pneumonia	Bacterial isolation (culture) * PCR ^1^	[63]
*Streptococcus equi* subsp. *equi*	Strangles	qPCR ^2,^* Bacterial isolation (culture) ELISA ^3^	[96]
*Taylorella equingenitalis*	Contagious equine metritis (CEM)	Bacterial isolation (culture) * IFAT ^4^ Real-time PCR	[110]
*Burkholderia mallei*	Glanders	CFT ^5,^* Bacterial isolation (culture) ELISA PCR	[127]

^1^ Polymerase chain reaction, ^2^ quantitative PCR, ^3^ enzyme-linked immunosorbent assay, ^4^ immunofluorescence antibody test, ^5^ complement fixation test. * Gold standard technique.

**Table 2:** Current loop-mediated isothermal amplification (LAMP) assays developed for equine bacterial disease of biosecurity importance.

Disease	Target Gene	Sample	Monitoring	Analytical Sensitivity	Clinical Sensitivity	Specificity	Reference
Contagious equine metritis	23s rRNA	Culture isolate, Genital swabs ^1^	Turbidity	24.8 copies/rxn	71%	100%	[161]
Glanders	Integrase	Culture isolate, Clinical isolate	Turbidity	22 ng/µL	NA ^^^	100%	[162]
	*BMA10229_375*	Culture isolate, Blood ^1^	Turbidity	1 pg/rxn	NA	100%	[46]
	*Flip*-IS*40J*A	Culture isolate, Blood ^1^	Turbidity	0.25 pg/rxn	NA	100%	[12]
Pneumonia	*vapA*	Clinical sample (TW) ^2^	Turbidity	10 CFU/rxn	91.4%	93.8%	[44]
Strangles	*seM*	Clinical sample (NW) ^3^	Turbidity	0.1 CFU/rxn	NA	100%	[163]
	*eqbE*	Clinical samples (NP FS ^4^, NPW ^5^, GPL ^6^)	Fluorescence	1 CFU/rxn	77%	78%	[154,164]
	*eqbE*	Clinical samples (GPL)	Microfluidic device	1 CFU/rxn	100%	62%	[45]

^1^ Spiked sample, ^2^ Tracheal wash, ^3^ Nasal wash, ^4^ Nasopharyngeal flocked swab, ^5^ Nasopharyngeal wash, ^6^ Guttural pouch lavage. ^^^ Result not reported.

## Data Availability

Data sharing not applicable.
